# CIELab Colour Coordinate Changes Associated with the Resolution of Gingival Inflammation: Influence of Biotype and Severity

**DOI:** 10.3390/jcm14134575

**Published:** 2025-06-27

**Authors:** Cristina Gómez Polo, Yasmina Guadilla, María Portillo Muñoz, María Lobato Carreño, Javier Flores Fraile, Norberto Quispe López, Ana María Martín Casado

**Affiliations:** 1 Department of Dentistry, School of Medicine, Campus Miguel de Unamuno s/n, University of Salamanca, 37007 Salamanca, Spain; yguadilla@usal.es (Y.G.); mportillo@usal.es (M.P.M.); mlobato@usal.es (M.L.C.); j.flores@usal.es (J.F.F.); norberto_quispe@usal.es (N.Q.L.); 2 Department of Statistics, School of Medicine, University of Salamanca, 37007 Salamanca, Spain; ammc@usal.es

**Keywords:** spectrophotometry, gingival diseases, subgingival curettage, inflammation, colour

## Abstract

**Background/Objectives:** The colour changes related to inflammation have yet to be quantified despite the fact that the intensification of gingival colour is one of the cardinal signs of gingival inflammation. We aimed to (1) assess the effect of periodontal treatment on gingival colour according to biotype and initial degree of inflammatory severity and (2) identify the relationship between the plaque index and gingival colour and compare the colour of moderately or severely inflamed gums to the colour of gums with mild inflammation. **Methods**: Forty-three participants with visual signs of inflammation were included in the study. CIELAB colour coordinates were measured using a spectrophotometer at the distal papilla, free gingival margin, middle zone of the attached gingiva, and mucogingival line proximate to the maxillary central incisor pre-treatment and four weeks post treatment with periodontal debridement. Each participant’s gingival biotype, plaque and gingival indices, age, and gender were also recorded. **Results**: The biotype and plaque index were both found to influence the colour of inflamed gums, albeit only the a* coordinate in two of the zones examined (the distal papilla and free gingival margin). Statistically significant differences were also found between the a* coordinates of participants with and without plaque at the distal papilla and the free gingival margin. The severity of inflammation also influenced the L* coordinates of gingival colour (*p* < 0.001) in all participants in all four zones examined. Periodontal treatment only affected the L* coordinate of gingival colour at the distal papilla and the free gingival margin. **Conclusions**: When gingival inflammation occurs, participants with thin biotype and plaque have redder gums, and moderately or severely inflamed gingiva have a visually perceptible darker colour. Periodontal treatment significantly increases the lightness of the distal papilla and the free gingival margin. Further, periodontal treatment creates a perceptible colour change in all zones under study, which is not dependent on either the patient’s biotype or initial severity of inflammation. Clinical Significance. Digital spectrophotometric analysis provides a valuable technological enhancement to quantify gingival colour and record changes in gingival colour during the course of periodontal treatment. Regardless of the initial severity of gingival inflammation, a perceptible increase in the L* coordinate (gums become visibly lighter) can be expected after periodontal treatment. Regardless of the initial severity of inflammation, a perceptible increase in the L* coordinate can be expected (gums become visibly lighter) after treatment.

## 1. Introduction

Gingivitis or gingival inflammation is a mild infectious form of periodontal disease, leading to irritation, redness, oedema, high temperature, bleeding on probing, and increased gingival bleeding, often caused by the accumulation of bacterial plaque or tartar [1]. Treating gingivitis in a timely manner is important, as it is reversible with professional treatment and improved outpatient hygiene measures (brushing teeth three times per day and using dental floss/interdental toothbrushes and antiseptic mouthwash). In the early stages, gingivitis is usually asymptomatic, which delays visits to the dentist. Untreated persistent gingivitis can progress to periodontitis, which is associated with the irreversible loss of attached periodontal tissues, including the teeth [1,2,3,4]. The most frequent cause of gingivitis is poor oral hygiene. There are two signs that are very often used to diagnose gingivitis, which are detected by visual examination and do not require invasive methods: intensification of gingival colour and increase in volume, beginning at the free gingival margin [5]. The severity of gingivitis depends on the host inflammatory response as well as the pathogenic potential and quantity of anaerobic Gram-negative bacteria present in the gingival biofilm [5]. Two of the main causes of gingival colour changes are vascular changes (the quantity, dilation, and permeability of vessels increase) and decreased keratinisation of the epithelium [5,6]. These underlying alterations are the primary drivers of the clinical colour changes described above. The gums usually become darker, redder/bluer, and bleed easily or spontaneously [5]. In contrast, healthy gums are firm, pale pink in colour, and well attached around the teeth [7]. Gingival colour changes can contribute to a diagnostic suspicion of clinical pathology, but their negative aesthetic impact should also be considered, particularly for patients with high smile lines [8,9,10,11,12]. Gingival diseases are among the most prevalent intraoral pathologies [12]. Gingivitis/periodontitis affects approximately 70% of the adult population at some point in their lives. Severe periodontal diseases are estimated to affect around 19% of adults, which translates to over a billion cases worldwide. [13]. The primary risk factors are poor oral hygiene and smoking. Plaque control by patients is therefore essential, meaning that motivating and instructing them on improved oral hygiene is a particularly important part of treatment, both in the early stages and the long term [14]. Smoking can cause smoker’s melanosis [15], an increased risk of gingival fibrosis [16], and reduced vascular density [17].

There is no clear consensus about the full range of variables that define gingival colour or the relative impact of each upon the colour of natural healthy gingiva [18]. Attempts have been made to quantify the influence made by variables such as gender, age, smoking habits, and frequency of toothbrushing, but such research has yet to produce firm or conclusive results [19]. Further, gingival colour depends on certain anatomical components whose influence has yet to be scientifically studied, the most important of which include vascular number and size, epithelial thickness, degree of keratinisation, and pigments in the gingival epithelium [20]. The main pigments that give the oral mucosa its colour are melanin, melanoid, carotene, reduced haemoglobin, and oxyhaemoglobin [20,21]. In almost all periodontal indices, colour is one of the main factors used to diagnose gingival conditions and determine their severity (with more marked colour changes indicating more severe periodontal pathology) [22,23,24,25,26]. Two such examples are the Sulcus Bleeding Index [27] and the Modified Gingival Index [28].

While the use of digital electronic devices to objectively measure colour has been established in dentistry for many years, gingival classifications and indices are still based on a limited number of categories using narrative description of colour. For example, the Gingival Index [22] classifies gums as having a normal colour, slight change in colour, redness, or marked redness. Such visual colour assessments are subjective, and publications using objective colour coordinates for this purpose are still lacking [12]. Gingival colour is evidently an important factor for determining the presence of gingival inflammation as well as the severity of symptoms. Despite this, a protocol has yet to be established for relating gingival colour coordinates to gingival biotype or inflammation severity, although several options for digital analysis of gingival colour have been explored [12,29,30]. Only a few studies have attempted to quantify colour changes in inflamed gingiva, and none of these have used a spectrophotometer, which is the preferred electronic device due to its superior reliability for gingival colour measurement [31]. Using spectrophotometry for colour analysis has the advantage of increased precision and sensitivity to colour changes, improving the likelihood of detecting small alterations in colour. Unlike in periodontology, this technology is already well established in the dental field for measuring changes in colour (recording efficacy) after dental whitening treatments [32].

Periodontal treatment with debridement provides many benefits to oral health: improving the aesthetics and functionality of teeth and gums [33], removing tartar and bacterial plaque [34], reducing the risk of mobility and loss of teeth [35], preventing halitosis [36,37], reducing the risk of cardiovascular disease [38] and bleeding gums [39,40], decreasing periodontal pocket depth, increasing clinical attachment levels, and reducing signs of inflammation such as oedema, texture, bleeding, and colour compared to pre-treatment levels [12]. Clinical examination through periodontal probing is used to ascertain the probing pocket depth and bleeding on probing, providing cross-sectional and longitudinal data on periodontal health, which supports diagnosis and follow-up of the disease. While periodontal indices are a valuable tool for diagnosing periodontal disease, the intra- and inter-observer reproducibility and consistency of the periodontal assessments made have been called into question [41,42,43,44]. Consequently, these indices may underestimate or overestimate the frequency and severity of periodontal conditions. These potential errors [42] could be eliminated through the use of objective methods such as gingival colour measurement with spectrophotometry, which was one of the motivations for conducting the present study. As described in narrative form by gingival indices, colour changes upon resolution of inflammation (when healthy gingival colour returns) are visually perceptible. Gingival colour usually becomes paler (redness is reduced) as inflammation goes down due to a decrease in vascularisation and increase in epithelial keratinisation, among other factors. In daily clinical practice, the methods used to quantify gingival colour are not appropriate, as they are often subjective, inaccurate, and difficult to communicate in a standardised way between professionals. This limitation represents a major challenge, especially considering that gingival colour is a basic and essential variable for the diagnosis of gingivitis. The absence of objective and reproducible tools hinders the reliable assessment of tissue changes, thus compromising the early detection and adequate follow-up of this periodontal disease.

The CIELAB colour system was developed by the CIE (Commission Internationale de l’Eclairage) in 1976 [45] with the aim of standardising colour measurement, and it has become internationally recognised as the standard colorimetric system [46]. The colour model contains three types of coordinates: an axis of L* coordinates ranges from black (0) to white (100), a* coordinates range from green (a* < 0) to red (a* > 0), and b* coordinates range from blue (b* < 0) to yellow (b* > 0). When it comes to quantifying colour differences, the following two formulae are most the commonly used in dentistry research [46]. The Euclidean formula was the standard means of calculating colour difference:

∆Eab = [(∆L*)2 + (∆a*)2 + (∆b*)2]^1/2^

The CIEDE2000 formula was developed by the CIE to better represent the visual perception of human observers [47,48,49,50]:

$${\left[{\left(\frac{∆L\prime }{K_{L}S_{L}}\right)}^{2}+{\left(\frac{∆C\prime }{K_{C}S_{C}}\right)}^{2}+{\left(\frac{∆H\prime }{K_{H}S_{H}}\right)}^{2}+R_{T}\left(\frac{∆C\prime }{K_{C}S_{C}}\right)\left(\frac{∆H\prime }{K_{H}S_{H}}\right)\right]}^{1/2}$$

Based on the research context described above, the authors developed the following objectives concerning the use of spectrophotometry in periodontology to quantify colour changes related to gingival inflammation processes. 

The objectives are (1) to assess the effect of biotype on the colour of inflamed gingiva, (2) to identify the relationship between the plaque index and gingival colour and compare the colour of moderately or severely inflamed gums to the colour of gums with mild inflammation, and (3) to assess the effect of periodontal treatment on gingival colour. The null hypotheses include the following: (1) The colour of inflamed gingiva in patients with a thin gingival biotype is no different from the colour of inflamed gingiva in patients with a thick gingival biotype. (2) There is not chromatic relationship between plaque index and gingival colour. The colour of moderately or severely inflamed gingiva is the same as that of mildly inflamed gingiva. (3) Periodontal treatment does not change the colour of the gingiva.

## 2. Materials and Methods

### 2.1. Patient Selection

All subjects above 18 years of age who attended the university’s dental clinic and met the following criteria were included: (1) visual signs of gingival inflammation: colour change and increase in volume (participants whose gingival colour change was perceptible to the operator—mainly increased redness), (2) no cognitive impairments, (3) without mucogingival surgery, (4) healthy natural tooth and gingival tissue in the aesthetic maxillary region, and (5) voluntarily acceptance of participation in the study. Participants were excluded who had systemic diseases and/or were taking long-term medication, were pregnant, had reduced upper-limb mobility, or had received orthodontic treatment. Also, participants with desquamative gingivitis or mucocutaneous conditions affecting the gingiva (such as lichen planus, pemphigoid, or pemphigus vulgaris) or recent use of antibiotics or anti-inflammatory drugs or smoking were excluded because these factors can independently influence gingival inflammation. The research protocol and study objectives were explained to each candidate. Participants who accepted inclusion in the study were required to sign the informed consent form. The research was approved by the university’s bioethics committee (BEC-594/21). The present study is non-randomised and longitudinal.

### 2.2. Collection of Data on Periodontal, Chromatic, and Hygiene-Related Variables

All data on periodontal, oral hygiene, and chromatic variables were collected by a single operator: a university lecturer in periodontology with over 20 years of experience (Y.G.G). Each participant sat in a standardised upright position in a chair during examination. Lip retractors were used to enable adequate illumination of the buccal cavity and clearly expose each participant’s teeth and gums. The surface of the mucosa was dried and cleaned using airflow or a gauze pad. Two photographs were taken per patient (before the initial treatment and after re-examination). 

In the first appointment, the following data were recorded at maxillary central incisor 11 or 21:

(1) The gingival biotype, using a probe to assess the transparency of the tissue [51];
(2) O’Leary’s Plaque Index [52], which is a dichotomous index (0/1) assessing the presence or absence of bacterial plaque at three sites vestibular and one site palatal to the cervical margin of the tooth. In the present research, only the vestibular sites were taken into consideration;
(3) Löe and Silness’ Gingival Index [22], using a CP-15 manual periodontal probe (Hu-Friedy, Chicago, United States). The Gingival Index developed by Löe and Silness [22] has four grades that relate bleeding on probing and change in gingival colour to the severity of inflammation. The scale is as follows:

Grade 0: Normal gingiva, no inflammation, no change in colour, no bleeding. 

Grade 1: Mild inflammation, slight change in colour, minimal superficial alterations, no bleeding. 

Grade 2: Moderate inflammation, redness, oedema, bleeding on probing and application of pressure. 

Grade 3: Severe inflammation, marked redness, oedema, tendency to spontaneous bleeding, potential ulceration;

(4) The colour coordinates obtained using spectrophotometry. After calibration of the SpectroShade Micro spectrophotometer (MHT Optic Research, Zürich, Switzerland), three colour measurements were made per participant, including both papillae and the mucogingival line at the zenith of the incisor. The colour coordinates (L*, a*, and b*) were measured in each of the four zones under study: the free gingival margin, middle zone of attached gingiva, mucogingival line, and distal papilla. The mean was used in the statistical analysis to minimise deviations. The spectrophotometer’s colour-measurement window was used to record these coordinates by tracing out a triangular shape that included the entire distal papilla and a rectangle with a scalloped edge extending in a cervical direction from the free gingival margin to the mucogingival line, where the coordinates of free gingival margin, middle zone of attached gingiva, and mucogingival line were collected. All three colour coordinates were measured at the distal papilla and in the vestibular gingiva, which was divided into three horizontal areas: the lower containing the free gingival margin, the middle area including the attached gingiva, and the upper containing the mucogingival line.

**Figure 1:**
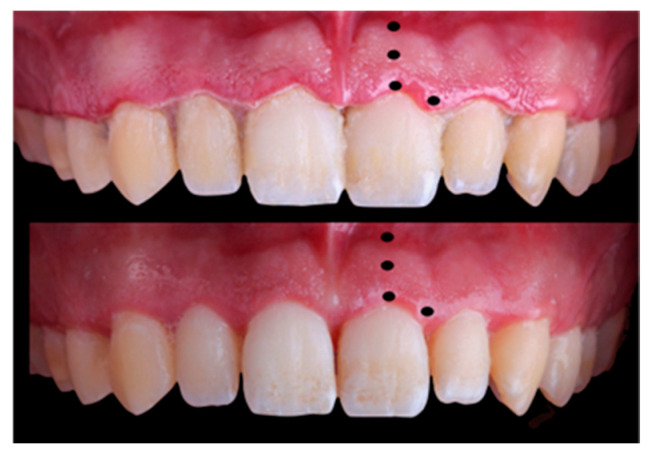
Gingival chromatic registration zones.

All these variables were recorded prior to treatment and then repeated four weeks after periodontal treatment [53], except the gingival biotype (Figure 1). In this first visit, scaling and root surface debridement was performed, including removal of calculus as well as supra- and subgingival biofilm. In addition, the principal investigator instructed participants on conventional and interdental toothbrushing, encouraging them to improve their dental hygiene. A verbal explanation was also given on the relationship between inflammation and dental plaque and how plaque can be removed through frequent and effective hygiene measures. 

### 2.3. Statistical Analysis

The statistical analysis produced basic descriptive statistics for the quantitative variables and frequencies and percentages for the qualitative variables. Comparisons were made using *t*-tests for paired and unpaired samples, the independent samples proportions test, and McNemar’s test. The effect sizes in the comparison of means tests were measured using Cohen’s d. (Cohen’s d values below 0.2 are considered negligible, values between 0.2 and 0.5 are interpreted as small, those between 0.5 and 0.8 as medium, and values above 0.8 as large effects.) The statistical significance level was set at 0.05. All analyses were performed using SPSS software (version 28). To assess the effect of the biotype and periodontal treatment on gingival colour, colour differences were calculated (using the Euclidean and CIEDE2000 formulae), then summarised using descriptive statistics, and compared with the published 50:50% perceptibility thresholds [54].

## 3. Results

A total of 43 participants took part in the study, all of Caucasian race, although this was not an inclusion criterion. The descriptive statistics for the total sample are shown in Table 1. 

For the first two study objectives, only data collected prior to treatment (baseline data) were used, while for the last two objectives, the baseline data were compared to the data collected four weeks after periodontal treatment.

### 3.1. Biotype—Gingival Colour

Table 2 shows the means and standard deviations of the colour coordinates recorded in participants with thin and thick biotypes.

The colour differences between the inflamed gingiva of the 450 pairs formed by one participant with a thin biotype and another with a thick biotype are summarised in Table 3.

Using both formulae, the percentage of differences that were perceptible (ΔEab > 3.1; ΔE00 > 2.1) [54] in the four gingival zones was over 90%, indicating that the biotype has a clinically significant effect on colour coordinates in inflamed gums. The colour of inflamed gingiva differed according to the participant’s gingival biotype, and the a* coordinate made the greatest contribution to this difference. Therefore, inflamed gums in participants with a thin biotype generally contain a larger quantity of red (a* coordinate).

### 3.2. Severity of Gingival Inflammation—Gingival Colour

The plaque index (indicating whether or not plaque is present) and the gingival index (moderate or severe inflammation/mild inflammation) were both used in this assessment of gingival inflammation. To evaluate the effect of the plaque index on colour, plaque was considered to be present when plaque was identified in all three sites examined (distal vestibule, vestibular, and mesial vestibule). Table 4 shows the effects of plaque on the colour of inflamed gums (bearing in mind that participants were only classed as having plaque when it was present in all three aforementioned sites). Those participants for whom plaque was recorded in one or two of the sites examined were included in the ‘no plaque’ category.

To assess the effect of the gingival index on colour, inflammation was considered to be moderate or severe when inflammation of that grade was recorded in at least two of the three sites examined (distal vestibule, vestibular, and mesial vestibule). Table 5 shows the effects of moderate or severe inflammation compared to mild inflammation on the colour of inflamed gingiva.

### 3.3. Effect of Periodontal Treatment on the Colour of Inflamed Gingiva

Table 6 shows the effect of periodontal treatment on gingival colour, according to gingival zone.

**Figure 2:**
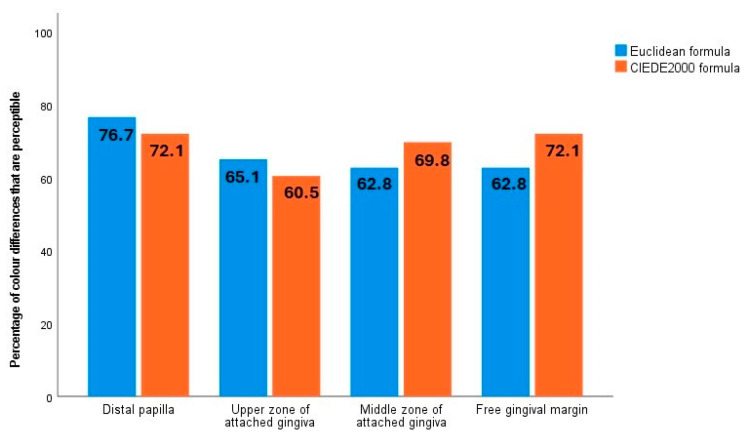
Percentage of colour differences that were perceptible in each gingival zone, according to the Euclidean and CIEDE2000 formulae.

Using both formulae, the percentage of perceptible differences (ΔEab > 3.1; ΔE00 > 2.1) [54] in the four gingival zones was above 60% (Figure 2), indicating that treatment has a clinically significant effect on colour coordinates. The largest differences between pre-treatment and post-treatment colour were observed at the distal papilla and the free gingival margin. 

**Figure 3:**
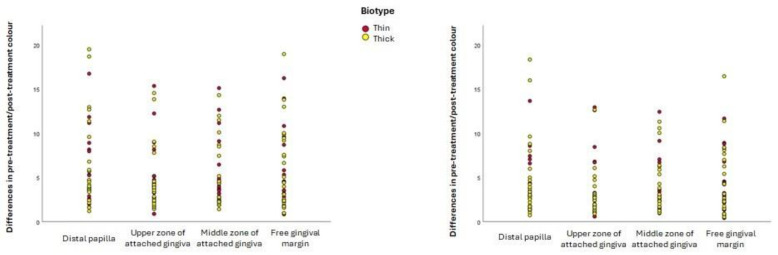
Differences in colour before and after treatment, according to biotype and gingival zone. Left: colour differences calculated using the Euclidean formula; right: colour differences calculated using the CIEDE2000 formula.

The change in the colour of inflamed gingiva that occurs after treatment does not depend on the patient’s biotype (Figure 3), as no statistically significant differences were found between the colour differences for participants with a thin biotype and those with a thick biotype in any of the gingival zones using either formula, as the following summary shows. Euclidean formula: distal papilla: t = 0.081, *p* = 0.936; upper zone of attached gingiva: t = 0.653, *p* = 0.518; middle zone of attached gingiva: t = 0.580, *p* = 0.565; free gingival margin: t = −0.043, *p* = 0.966. CIEDE2000 formula: distal papilla: t = −0.139, *p* = 0.890; upper zone of attached gingiva: t = 0.497, *p* = 0.622; middle zone of attached gingiva: t = 0.291, *p* = 0.773; free gingival margin: t = −0.225, *p* = 0.823.

**Figure 4:**
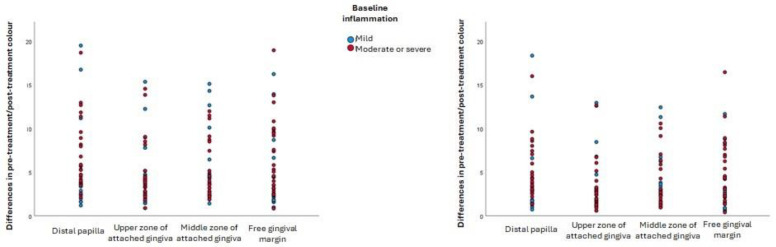
Differences in colour before and after treatment, according to severity of inflammation and gingival zone. **Left**: colour differences calculated using the Euclidean formula; **right**: colour differences calculated using the CIEDE2000 formula.

The colour change that occurs in inflamed gums after periodontal treatment is also independent of the baseline severity of inflammation (Figure 4). No statistically significant differences were found between the colour differences for participants whose initial inflammation was moderate or severe and participants whose baseline inflammation was mild in any gingival zone using either of the two formulae, as summarised in the following data. Euclidean formula: distal papilla: t = 1.091, *p* = 0.282; upper zone of attached gingiva: t = −0.498; *p* = 0.621; middle zone of attached gingiva: t = 0.819, *p* = 0.418; free gingival margin: t = −1.279, *p* = 0.208. CIEDE2000 formula: distal papilla: t = −0.836, *p* = 0.408; upper zone of attached gingiva: t = -0.336, *p* = 0.739; middle zone of attached gingiva: t = 0.404, *p* = 0.688; free gingival margin: t = −1.793, *p* = 0.080.

## 4. Discussion

Gingivitis and periodontal disease are, together with caries, among the most common oral pathologies in humans. These infectious conditions progressively damage the periodontium, potentially leading to loss of teeth [3,4] and significantly reducing quality of life [55]. Conventionally, inflammation is identified using Celso’s four cardinal signs [19]: calor (heat), rubor (redness), tumor (swelling/oedema), and dolor (pain). Calor and rubor occur because of the vascular changes that lead to an accumulation of blood in the affected area. The healing process after root surface debridement can involve repair or regeneration, which can affect the appearance and quality of the tissue [5,56]. Objectively determining the colour changes resulting from inflammation and its subsequent resolution is therefore vital, but subjective evaluation of such chromatic changes continues to be standard practice. Quantifying gingival colour changes through spectrophotometry is a precise, non-invasive means to support early diagnosis of alterations to the gingival tissue [19] and to objectively assess the chromatic response of the gingiva to periodontal treatment. This methodological improvement has yet to be studied in depth, and the definition of gingival colour is a field that continues to require further research. The objectives of the present study were designed to respond to these research gaps as well as addressing the need to quantify the colour change caused by gingival inflammation and determine its relationship to biotype, plaque, and periodontal treatment. 

All the null hypotheses of the present research should be rejected. The first should be rejected because statistically significant differences were found between the a* coordinates of participants with thin biotypes and thick biotypes: the former had perceptibly redder inflamed gingiva than the latter. The second null hypothesis should be rejected because moderately or severely inflamed gingiva were darker than mildly inflamed gingiva in all gingival zones examined. The third null hypothesis should be rejected since debridement created a colour change in all gingival zones examined, which in most cases was perceptible: there were statistically significant changes in the L* coordinate (lightness) at the free gingival margin and the distal papilla (lightness), where the gingiva became lighter (higher L* coordinates) after inflammation was resolved. The colour change after resolution of gingival inflammation was independent of the initial severity of inflammation. 

These findings are limited to a Caucasian population and need to be corroborated by further longitudinal research that includes several follow-ups over a longer time period, is applied to different racial populations [57], and uses larger sample sizes. Other potential issues, such as the lack of blinding, intra-examiner error, short follow-up period (four weeks), or potential measurement errors with the spectrophotometer, could have influenced the results obtained. It would also be useful to explore the confidence intervals of the colour coordinates corresponding to each category in periodontal disease classification systems. Such research could help determine chromatic ranges to support diagnosis, with gingival colour data providing clinicians with information about patients’ periodontal health.

Existing periodontal indices [58] describe colour changes using a range of narrative descriptions (such as ‘normal-coral colour’, ‘slight change of colour’, ‘redness’, and ‘marked redness’) whose utility and precision is limited by disparities between the subjective chromatic perception of distinct observers. Improving the classification and diagnosis of periodontal pathologies through standardised, objective quantification of gingival colour is not possible through the use of such instruments alone. In an attempt to eliminate subjectivity and inter- or intra-observer inconsistencies from colour selection [59,60], non-invasive methods for diagnosing periodontal disease based on digital technologies are starting to appear [26]. These methods primarily measure volumetric [61,62,63] or colour [12,30,64] changes. More work is needed to improve diagnostic precision and to design well-targeted, reliable treatment plans [65]. Informing patients about positive aesthetic changes resulting from treatment, perhaps by showing them the estimated colour of healthy gums after debridement, could increase motivation for treatment requests and adherence thereto. Additionally, it is widely recognised that such aesthetic improvements have positive psychosocial repercussions for patients [66].

### Strengths and Limitations of the Study 

To date, the present study is the first that has used spectrophotometry to precisely quantify colour changes in gingiva during and after inflammation. The main advantages of current spectrophotometers developed for use in dentistry are their objectivity and operator independence, along with high levels of temporal stability and reliability [31]. Most of these devices were designed to measure dental colour and provide results either in colour coordinates or standard shade-guide terminology (Vita Classical/3D Master). Only a limited number of spectrophotometer models have been used in gingival research [19,67], and their physical design prevents their use in posterior regions because the ‘open window screens’ used for colour measurement are too big to fit inside the mouth or to enable correct alignment with premolars or molars. For practical clinical purposes, then, the use of objective colour measurement to support diagnosis has been limited to the anterior region, and in general, spectrophotometry has yet to make a significant impact on everyday diagnostic practice. 

The present study had a similar sample size to that used in related longitudinal studies on inflamed gingiva before and after periodontal treatment [12,30]. These earlier studies by Mayer et al. in 2017 [30] and Ginesin et al. five years later [12] used photographs from a digital single-lens-reflex (DSLR) camera to record colour changes after debridement. However, this necessitated conversion of the colour in the photographs to colour coordinates using Adobe Photoshop CS4 software, as CIELAB coordinates could not be obtained directly from the patients using this method. This conversion process can introduce errors and imprecision into the data. Another factor that can affect the chromatic results is the zone in which colour is measured. Mayer et al. measured a gingival band extending from the mesial to the distal papilla adjacent to the maxillary lateral incisor, including the free gingiva, keratinised gingiva, and part of the papilla proximate to this incisor [30], whereas most of Ginesin et al.’s colour measurements were in posterior regions of the maxilla and mandible [12]. It may be suspected that there is a homogeneous response to the resolution of inflammation across the intraoral regions, but we cannot make any such claim unequivocally in the absence of more complete spectrophotometric data. Further, the reasonable assumption that toothbrushing may be less vigorous in non-visible zones would also cast doubt on such a statement. The aforementioned authors analysed the mean colour coordinates of the whole selected area, which makes the real chromatic effect of each anatomical zone of the gingiva less clear since the singular characteristics of each region may create a distinct chromatic response. In the present research, a spectrophotometer was used to make direct colour measurements of each anatomical region [68] in order to perform a more precise, fine-grained assessment of how the resolution of inflammation affects the gingiva adjacent to the maxillary central incisor. It should be noted that some research [69,70,71] has found significant correlations between the colour measurements resulting from digital photography and from spectrophotometry. Like the present research, both of the studies described above [12,30] included participants with different types of periodontal diagnoses [58,72], thereby providing an overarching vision of the resolution of gingival inflammation. 

In inflamed gingiva, the present findings show changes in the a* coordinate to be of greatest importance, with the values of this coordinate being higher in subjects with plaque than participants without plaque at the distal papilla (28.0 vs. 25.2) and the free gingival margin (28.1 vs. 24.7). The intensity of redness is therefore greater in patients with an accumulation of plaque, particularly in the zones located closest to the bacterial plaque, which is where the inflammation begins. The mean initial a* coordinate in Mayer et al. and Ginesin et al.’s studies (approximately 32.0) [12,30] for participants with distinct types of inflammation was greater than the mean initial values for this coordinate presented here, which could be due to the methodological differences explained above. 

In all four gingival zones under study, the gingiva with the most severe initial inflammation were found to be darker (lower L* value), while no statistically significant differences in the a* coordinate were identified. This finding was also evident in the effect observed after inflammation was resolved. The main difference between the present research and similar studies [12,30] was that it found lightness (L*) to be the only coordinate to have undergone statistically significant changes four weeks after root surface debridement. Again, the free gingival margin (48.8 vs. 50.7) and the distal papilla (46.3 vs. 48.2) were the sites of these changes, where the gingiva became lighter. Other studies [12,30] have determined the a* coordinate to be responsible for most of the colour change after inflammation is resolved, with the gingiva becoming less red. Nevertheless, Ginesin et al. [12] also found statistically significant changes in the L* coordinate before and after treatment (56.3 vs. 60.7). From another perspective, the results of all the studies discussed concur in that the colour changes presented were perceptible to the human eye [12,30]. However, the authors of the earlier studies only used the Euclidean formula [12,30] and referred to perceptibility thresholds created for the dental colour space, whereas use of thresholds designed for the gingival colour space would be preferable to ensure accurate interpretations [54]. In the present study, the percentage of pre- and post-treatment colour differences that were perceptible was above 60% in all four zones examined, using both formulae (ΔEab > 3.1 and ΔE00 > 2.1 thresholds specific to the gingival colour space). 

The fact that colour change after debridement treatment did not depend on either the initial severity of inflammation or the gingival biotype is particularly interesting. This novel finding arose from analysing inflammation and gingival biotype from a chromatic perspective since few studies have explored this topic recently with spectrophotometry [73] or with objective methodology like intraoral scanners [29,74,75]. 

One of these studies assessed the consistency between gingival inflammation scores obtained directly using the modified gingival index (MGI) and those derived from intraoral scans (IOS) evaluated 10 days later. Conducted on 23 healthy adults aged 18–72, the study analysed 552 gingival sites. The findings showed a 90% agreement between chairside and IOS-based MGI scores, with the most frequent discrepancies involving minor one-point differences. Scores derived from IOS were consistent across two evaluators, with 91% agreement and no variations exceeding one scale point. The results confirm that intraoral scans reliably capture gingival characteristics such as contour and colour, allowing accurate inflammation assessment. [29]. Intraoral scanning proves to be a reliable, non-invasive tool for evaluating gingival health, offering significant potential for clinical practice and remote diagnostics through accurate data collection and analysis. [29]. Along the same lines is a later publication in which digital colour analysis of intraoral scans showed promising results in identifying sites with bleeding on probing (BOP), ranging from acceptable to excellent performance. This technology represents a valuable tool for improving the detection and management of gingivitis [74]. In our study, we investigated a more objective method for identifying gingival inflammation by analysing colour data from intraoral scans, reducing reliance on the subjective visual assessment typically used by clinicians. We evaluated 110 scans from 55 individuals, comparing gum colour before and after treatment. Using specific colour metrics, we were able to differentiate between healthy and inflamed tissues with up to 80.8% accuracy. These results are very interesting, but they do not use the ‘gold standard’ methodology for recording colour in dentistry, nor do they offer results based on the severity of inflammation or gingival biotype.

A systematic review suggested that although the use of artificial intelligence to detect dental plaque, diagnose gingivitis, and measure alveolar bone loss is still under development, these technologies have the potential to become valuable tools for clinical support and diagnosis [75]. Another similar study, whose results cannot be directly compared with the previous ones, is the one published by Shrivastava et al., as it extracted colour coordinates from intraoral photographs and did not record them in vivo [76]. 

Inflamed gingiva with a thin biotype were found to have a larger quantity of red at the free gingival margin and the distal papilla than inflamed gingiva with a thick biotype. Although the underlying cause remains uncertain, this may be related to the degree of keratinisation of the epithelium [77,78]. The colour differences in the inflamed gums of participants with distinct biotypes were clearly visible, exceeding the thresholds of clinical acceptability [54]. It should be noted that Mayer et al.’s study [30] did not identify significant changes after periodontal treatment in either the severe periodontitis or gingivitis group, although the samples used were not large (the maximum sample size was 19), and the signs and symptoms of gingivitis were rather variable [28]. All this suggests that the chromatic gingival response after debridement is complex and may have several influences, including genetic and hormone-related factors, smoking, and oral hygiene habits [19]. Further, not only can plaque accumulation can cause colour changes [79] but also nodular gingival lesions [80] such as pyogenic granulomas [79] and the presence of dental implants with thin gingival biotypes [67].

Digital colour analysis may therefore be useful as a diagnostic aid, as it is more sensitive than the human eye, enabling it to detect the minor chromatic changes related to such phenomena. One of the impediments to the incorporation of spectrophotometry into daily clinical practice is its high cost. Spectrophotometry, which would be useful in restorative dentistry, tooth whitening, caries diagnosis, material ageing, and periodontal diagnosis, is practically used for research. It would be desirable in the coming years to expand its use to optimise treatment and diagnostic results. In the future, applications using artificial intelligence to analyse large datasets representing a significant proportion of the population may support clinicians from a diagnostic and motivational (increased treatment adherence) perspective since one of the first warning signs of periodontal pathology is colour change, and gums that are darker and redder are viewed less positively.

## 5. Conclusions 

With the limitations of this study, the following can be concluded: 

Periodontal therapy induces a significant increase in gingival lightness, particularly at the distal papilla and the free gingival margin. The resulting colour change is clinically perceptible in more than 60% of subjects across all four gingival regions assessed. Notably, this chromatic alteration appears to be independent of the patient’s periodontal biotype and the initial degree of inflammatory severity. There is a direct relationship between the level of plaque present and the redness (increased a* coordinate) and darkening (decreased L* coordinate) of the gingiva. These chromatic changes are evident.

## Figures and Tables

**Table 1:** Sociodemographic, oral hygiene, and clinical data on the study participants prior to treatment.

Sociodemographic Data		
Age		
	Range	20–79 years
	Mean (SD)	46.5 (46.2)
Gender		
	Women	23 (53.5%)
	Men	20 (46.5%)
Biotype		
	Thin	18 (41.9%)
	Thick	25 (58.1%)
**Oral hygiene data**		
Frequency of toothbrushing		
	Range	0–5 times/day
	Mean (SD)	2.1 (1.0)
Use of dental floss*		
	Never or hardly ever	38 (88.4%)
	Frequently	5 (11.6%)
Use of mouthwash *		
	Never or hardly ever	35 (81.4%)
	Frequently	8 (18.6%)
Interdental toothbrushing *		
	Never or hardly ever	37 (86.0%)
	Frequently	6 (14.0%)
**Clinical data**		
Plaque index: distal vestibule		
	No plaque	7 (16.3%)
	Plaque	36 (83.7%)
Plaque index: vestibular		
	No plaque	13 (30.2%)
	Plaque	30 (69.8%)
Plaque index: mesial vestibule		
	No plaque	7 (16.3%)
	Plaque	36 (83.7%)
Gingival index: distal vestibule		
	Mild inflammation	15 (34.9%)
	Moderate or severe inflammation	28 (65.1%)
Gingival index: vestibular		
	Mild inflammation	20 (46.5%)
	Moderate or severe inflammation	23 (53.5%)
Gingival index: mesial vestibule		
	Mild inflammation	14 (32.6%)
	Moderate or severe inflammation	29 (67.4%)

* Hardly ever: less than once per week; frequently: once per week or more.

**Table 2:** Mean colour coordinates (SD) according to zone of inflamed gingiva and biotype (coordinates that differ significantly are indicated in bold).

	Thin (n = 18)			Thick (n = 25)		
	L*	a*	b*	L*	a*	b*
Distal papilla	46.8 (6.3)	**28.7 (4.1)**	15.2 (2.2)	46.1 (5.3)	**26.0 (3.9)**	15.2 (1.8)
Upper zone of attached gingiva	48.8 (5.2)	27.2 (5.2)	16.5 (2.5)	49.2 (4.8)	25.5 (2.4)	15.2 (2.1)
Middle zone of attached gingiva	50.4 (5.4)	26.9 (4.7)	16.9 (2.0)	49.6 (5.0)	25.9 (3.4)	15.6 (2.2)
Free gingival margin	49.6 (6.3)	**28.7 (4.9)**	17.0 (1.9)	48.2 (6.5)	**25.9 (3.7)**	16.5 (1.9)

Significant differences were found between the a* coordinates of inflamed gingiva in participants with a thin biotype and those with a thick biotype at the distal papilla (28.7 vs. 25.9, t = 2.151, *p* = 0.019, Cohen’s d = 0.665): inflamed gingiva in participants with a thin biotype were redder than in participants with a thick biotype.

**Table 3:** Basic descriptive statistics on the colour differences between inflamed gingiva in each pair of participants with a thin gingival biotype and thick biotype (n = 450), according to formula and gingival zone.

		Mean	SD	Minimum	Maximum
Euclidean formula					
Distal papilla		9.09	5.22	0.91	33.96
Upper zone of attached gingiva		8.47	4.73	1.29	26.48
Middle zone of attached gingiva		8.47	4.97	0.72	29.98
Free gingival margin		9.88	5.83	0.64	39.58
CIEDE2000 formula					
Distal papilla		6.77	4.76	0.45	33.49
Upper zone of attached gingiva		6.38	3.93	0.74	24.78
Middle zone of attached gingiva		6.63	4.18	0.58	23.29
Free gingival margin		7.65	5.14	0.55	37.25

**Table 4:** Mean colour coordinates (SD) according to zone of inflamed gingiva and plaque index (coordinates that differ significantly are indicated in bold).

	Plaque (n = 30)			No Plaque (n = 13)		
	L*	a*	b*	L*	a*	b*
Distal papilla	46.3 (5.9)	**28.0 (3.9)**	15.0 (2.1)	46.5 (5.4)	**25.2 (4.1)**	15.7 (1.6)
Upper zone of attached gingiva	48.6 (5.0)	26.8 (4.2)	15.7 (2.7)	49.9 (4.9)	24.9 (2.8)	15.9 (1.7)
Middle zone of attached gingiva	49.7 (5.0)	27.0 (3.8)	16.0 (2.2)	50.5 (5.7)	24.8 (4.2)	16.4 (2.0)
Free gingival margin	48.8 (5.8)	**28.1 (4.2)**	16.7 (2.0)	48.8 (7.8)	**24.7 (4.1)**	16.8 (1.8)

Significant differences were only found between the a* coordinates of participants with and without plaque at the distal papilla (28.0 vs. 25.2, t = 2.097, *p* = 0.042, Cohen’s d = 0.696) and the free gingival margin (28.1 vs. 24.7, t = 2.399, *p* = 0.021, Cohen’s d = 0.796). In these gingival zones, participants with plaque had redder gums than those without plaque.

**Table 5:** Mean colour coordinates (SD) according to zone of inflamed gingiva and severity of inflammation (coordinates that differ significantly are indicated in bold).

	Moderate or Severe Inflammation			Mild Inflammation		
		(n = 27)		(n = 16)		
	L*	a*	b*	L*	a*	b*
Distal papilla	**44.1 (6.2)**	27.6 (4.2)	15.1 (1.7)	**50.1 (6.2)**	26.4 (4.0)	15.4 (2.5)
Upper zone of attached gingiva	**47.2 (3.7)**	26.2 (4.0)	15.5 (2.6)	**52.0 (5.3)**	26.2 (3.7)	16.1 (1.9)
Middle zone of attached gingiva	**47.5 (3.6)**	27.0 (3.9)	16.1 (2.4)	**54.0 (4.8)**	25.2 (3.9)	16.2 (1.6)
Free gingival margin	**46.1 (5.2)**	27.6 (4.3)	16.8 (2.1)	**53.4 (5.7)**	26.1 (4.6)	16.5 (1.7)

Statistically significant differences were identified between the L* coordinates of participants with moderate or severe inflammation and participants with mild inflammation in all four gingival zones (distal papilla: t = 3.810, *p* < 0.001, Cohen’s d = 1.100; upper zone of attached gingiva: t = 3.486, p = 0.001, Cohen’s d = 1.021; middle zone of attached gingiva: t = 5.075, *p* < 0.001, Cohen’s d = 1.601; free gingival margin: t = 4.333, *p* < 0.001, Cohen’s d = 1.367). In all gingival zones, participants with moderate or severe inflammation had darker gums than participants with mild inflammation. No statistically significant changes were detected in the a* and b* coordinates.

**Table 6:** Mean colour coordinates (SD) before and after periodontal treatment, according to the zone of inflamed gingiva (coordinates that differ significantly are indicated in bold), n = 43.

	Pre-Treatment			Post-Treatment		
	L*	a*	b*	L*	a*	b*
Distal papilla	**46.3 (5.7)**	27.2 (4.1)	15.2 (2.0)	**48.2 (4.0)**	26.5 (2.5)	15.5 (2.2)
Upper zone of attached gingiva: mucogingival line	49.0 (4.9)	26.2 (3.9)	15.8 (2.4)	49.7 (3.5)	26.2 (3.4)	15.6 (2.1)
Middle zone of attached gingiva	49.9 (5.2)	26.4 (4.0)	16.1 (2.2)	49.9 (3.9)	26.6 (3.4)	15.6 (2.2)
Free gingival margin	**48.8 (6.4)**	27.1 (4.4)	16.7 (1.9)	**50.7 (3.5)**	26.5 (3.4)	16.9 (2.3)

Statistically significant differences were found between the L* coordinates of participants before and after treatment at the distal papilla (46.3 vs. 48.2, *t*-test for paired samples: t = −2.258, *p* = 0.029, Cohen’s d = -0.344) and the free gingival margin (48.8 vs. 50.7, *t*-test for paired samples: t = −2.483, *p* = 0.017, Cohen’s d = −0.379). Periodontal treatment therefore made the gingiva lighter at the distal papilla and the free gingival margin, whereas almost no changes were observed in the other zones. The differences in colour pre- and posttreatment are summarised in Table 7.

**Table 7:** Basic descriptive statistics on colour differences in inflamed gingiva before and after periodontal treatment (n = 43), according to formula and gingival zone.

		Mean	SD	Minimum	Maximum
Euclidean formula					
Distal papilla		6.07	4.68	1.19	19.50
Upper zone of attached gingiva		4.73	3.68	0.88	15.35
Middle zone of attached gingiva		5.20	3.79	1.41	15.11
Free gingival margin		5.73	4.55	0.83	18.96
CIEDE2000 formula					
Distal papilla		4.51	3.97	0.72	18.34
Upper zone of attached gingiva		3.41	3.13	0.59	12.94
Middle zone of attached gingiva		3.77	3.05	0.95	12.43
Free gingival margin		4.30	3.59	0.40	16.45

## Data Availability

The original contributions presented in this study are included in the article. Further inquiries can be directed to the corresponding author(s).

## References

[1] James P., Worthington H.V., Parnell C., Harding M., Lamont T., Cheung A., Whelton H., Riley P., Cochrane Oral Health Group (2017). Chlorhexidine mouthrinse as an adjunctive treatment for gingival health. Cochrane Database Syst. Rev..

[2] Liu J., Liu Z., Huang J., Tao R. (2022). Effect of probiotics on gingival inflammation and oral microbiota: A meta-analysis. Oral Dis..

[3] Eklund S.A., Burt B.A. (1994). Risk Factors for Total Tooth Loss in the United States; Longitudinal Analysis of National Data. J. Public Health Dent..

[4] Takala L., Utriainen P., Alanen P. (1994). Incidence of edentulousness, reasons for full clearance, and health status of teeth before extractions in rural finland. Community Dent. Oral Epidemiol..

[5] Murakami S., Mealey B.L., Mariotti A., Chapple I.L.C. (2018). Dental plaque–induced gingival conditions. J. Periodontol..

[6] Chapple I.L.C., Van Der Weijden F., Doerfer C., Herrera D., Shapira L., Polak D., Madianos P., Louropoulou A., Machtei E., Donos N. (2015). Primary prevention of periodontitis: Managing gingivitis. J. Clin. Periodontol..

[7] Ciancio S., Panagakos F. (2010). Superior management of plaque and gingivitis through the use of a triclosan/copolymer dentifrice. J. Clin. Dent..

[8] Harb Z.K.J., El-Sayed W., Alkhabuli J., Pagano S. (2021). Gingival Depigmentation Using Diode 980 nm and Erbium-YAG 2940 nm Lasers: A Split-Mouth Clinical Comparative Study. Int. J. Dent..

[9] Altayeb W., Hamadah O., Alhaffar B.A., Abdullah A., Romanos G. (2021). Gingival depigmentation with diode and Er,Cr:YSGG laser: Evaluating re-pigmentation rate and patient perceptions. Clin. Oral Investig..

[10] Beşiroğlu-Turgut E., Kayaaltı-Yüksek S. (2023). Comparison of Er,Cr:YSGG laser and diode laser in the treatment of gingival melanin pigmentation: A randomized clinical trial. Lasers Med. Sci..

[11] Serio F.G., Strassler H.E. (1989). Periodontal and Other Soft Tissue Considerations in Esthetic Dentistry. J. Esthet. Restor. Dent..

[12] Ginesin O., Zigdon-Giladi H., Gabay E., Machtei E.E., Mijiritsky E., Mayer Y. (2022). Digital photometric analysis of gingival response to periodontal treatment. J. Dent..

[13] World Health Organization, Oral Health. https://www.who.int/news-room/fact-sheets/detail/oral-health.

[14] Heitz-Mayfield L.J., Lang N.P. (2013). Surgical and nonsurgical periodontal therapy. Learned and unlearned concepts. Periodontology 2000.

[15] Müller S. (2010). Melanin-associated pigmented lesions of the oral mucosa: Presentation, differential diagnosis, and treatment. Dermatol. Ther..

[16] Scott D.A., Singer D.L. (2004). Suppression of overt gingival inflammation in tobacco smokers—clinical and mechanistic considerations. Int. J. Dent. Hyg..

[17] Rezavandi K., Palmer R.M., Odell E.W., Scott D.A., Wilson R.F. (2002). Expression of ICAM-1 and E-selectin in gingival tissues of smokers and non-smokers with periodontitis. J. Oral Pathol. Med..

[18] Gómez-Polo C., Montero J., Gómez-Polo M., Casado A.M.M. (2019). Clinical study on natural gingival color. Odontology.

[19] Gómez-Polo C., Montero J., Casado A.M. (2024). Explaining the colour of natural healthy gingiva. Odontology.

[20] Sheel V., Purwar P., Dixit J., Rai P. (2015). Ancillary role of vitamin C in pink aesthetics. BMJ Case Rep..

[21] Ribeiro F.V., Cavaller C.P., Casarin R.C.V., Casati M.Z., Cirano F.R., Dutra-Corrêa M., Pimentel S.P. (2014). Esthetic treatment of gingival hyperpigmentation with Nd:YAG laser or scalpel technique: A 6-month RCT of patient and professional assessment. Lasers Med. Sci..

[22] Löe H. (1967). The Gingival Index, the Plaque Index and the Retention Index Systems. J. Periodontol..

[23] Polson A.M., Goodson J.M. (1985). Periodontal Diagnosis: Current Status and Future Needs. J. Periodontol..

[24] Lobene R.R., Weatherford T., Ross N.M., Lamm R.A., Menaker L. (1986). A modified gingival index for use in clinical trials. Clin. Prev. Dent..

[25] Bessa Rebelo M.A., Corrêa de Queiroz A., Panagakos F.S., Davies R.M. (2011). Gingival Indices: State of Art. Gingival Diseases—Their Aetiology, Prevention and Treatment.

[26] Trombelli L., Farina R., Silva C.O., Tatakis D.N. (2018). Plaque-induced gingivitis: Case definition and diagnostic considerations. J. Periodontol..

[27] Mühlemann H.R., Son S. (1971). Gingival sulcus bleeding--a leading symptom in initial gingivitis. Helv Odontol Acta..

[28] Trombelli L., Tatakis D.N., Scapoli C., Bottega S., Orlandini E., Tosi M. (2004). Modulation of clinical expression of plaque-induced gingivitis. II. Identification of “high-responder” and “low-responder” subjects. J. Clin. Periodontol..

[29] Daly S., Seong J., Parkinson C., Newcombe R., Claydon N., West N. (2021). A proof of concept study to confirm the suitability of an intra oral scanner to record oral images for the non-invasive assessment of gingival inflammation. J. Dent..

[30] Mayer Y., Ginesin O., Machtei E.E. (2017). Photometric CIELAB Analysis of the Gingiva: A Novel Approach to Assess Response to Periodontal Therapy. J. Periodontol..

[31] Sala L., Carrillo-De-Albornoz A., Martín C., Bascones-Martínez A. (2015). Factors involved in the spectrophotometric measurement of soft tissue: A clinical study of interrater and intrarater reliability. J. Prosthet. Dent..

[32] Frank A.C., Kanzow P., Rödig T., Wiegand A. (2022). Comparison of the Bleaching Efficacy of Different Agents Used for Internal Bleaching: A Systematic Review and Meta-Analysis. J. Endod..

[33] Kwon T., Lamster I.B., Levin L. (2021). Current Concepts in the Management of Periodontitis. Int. Dent. J..

[34] Radu C.-M., Radu C.C., Arbănaşi E.-M., Hogea T., Murvai V.R., Chiș I.-A., Zaha D.C. (2024). Exploring the Efficacy of Novel Therapeutic Strategies for Periodontitis: A Literature Review. Life.

[35] Graziani F., Karapetsa D., Alonso B., Herrera D. (2017). Nonsurgical and surgical treatment of periodontitis: How many options for one disease?. Periodontology 2000.

[36] Ortiz V., Filippi A. (2021). Halitosis. Monogr Oral Sci..

[37] De Geest S., Laleman I., Teughels W., Dekeyser C., Quirynen M. (2016). Periodontal diseases as a source of halitosis: A review of the evidence and treatment approaches for dentists and dental hygienists. Periodontology 2000.

[38] Orlandi M., Graziani F., D’AIuto F. (2020). Periodontal therapy and cardiovascular risk. Periodontology 2000.

[39] Di Stefano M., Polizzi A., Santonocito S., Romano A., Lombardi T., Isola G. (2022). Impact of Oral Microbiome in Periodontal Health and Periodontitis: A Critical Review on Prevention and Treatment. Int. J. Mol. Sci..

[40] Aimetti M. (2014). Nonsurgical periodontal treatment. Int J Esthet Dent..

[41] Hefti A.F., Preshaw P.M. (2012). Examiner alignment and assessment in clinical periodontal research. Periodontology 2000.

[42] Hill E.G., Slate E.H., Wiegand R.E., Grossi S.G., Salinas C.F. (2006). Study Design for Calibration of Clinical Examiners Measuring Periodontal Parameters. J. Periodontol..

[43] Mojon P., Chung J.P., Favre P., Budtz-Jörgensen E. (1996). Examiner agreement on periodontal indices during dental surveys of elders. J. Clin. Periodontol..

[44] Kingman A., Löe H., Ånerud Å., Boysen H. (1991). Errors in Measuring Parameters Associated With Periodontal Health and Disease. J. Periodontol..

[45] International Commission on Illumination (1976). Annuaire, Roster, Register, Annexe au Bulletin CIE.

[46] Technical Committee 1-48 (2004). Colorimetry CIE Publication No. 15.3.

[47] Gómez-Polo C., Muñoz M.P., Luengo M.C.L., Vicente P., Galindo P., Casado A.M.M. (2016). Comparison of the CIELab and CIEDE2000 color difference formulas. J. Prosthet. Dent..

[48] Paravina R.D., Ghinea R., Herrera L.J., Bona A.D., Igiel C., Linninger M., Sakai M., Takahashi H., Tashkandi E., Perez M.d.M. (2015). Color Difference Thresholds in Dentistry. J. Esthet. Restor. Dent..

[49] Pérez M.M., Ghinea R., Herrera L.J., Carrillo F., Ionescu A.M., Paravina R.D. (2018). Color difference thresholds for computer-simulated human Gingiva. J. Esthet. Restor. Dent..

[50] Gómez-Polo C., Montero J., Gómez-Polo M., Casado A.M. (2020). Comparison of the CIELab and CIEDE 2000 Color Difference Formulas on Gingival Color Space. J. Prosthodont..

[51] Malpartida-Carrillo V., Tinedo-Lopez P.L., Guerrero M.E., Amaya-Pajares S.P., Özcan M., Rösing C.K. (2021). Periodontal phenotype: A review of historical and current classifications evaluating different methods and characteristics. J. Esthet. Restor. Dent..

[52] O’Leary T.J., Drake R.B., Naylor J.E. (1972). The Plaque Control Record. J. Periodontol..

[53] Morrison E.C., Ramfjord S.P., Hill R.W. (1980). Short-term effects of initial, nonsurgical periodontal treatment (hygienic phase). J. Clin. Periodontol..

[54] Gómez-Polo C., Casado A.M.M., Gómez-Polo M., Montero J. (2020). Colour thresholds of the gingival chromatic space. J. Dent..

[55] Wong L.B., Yap A.U., Allen P.F. (2021). Periodontal disease and quality of life: Umbrella review of systematic reviews. J. Periodontal Res..

[56] Wikesjö U.M., Selvig K.A. (1999). Periodontal wound healing and regeneration. Periodontology 2000.

[57] Ho D.K., Ghinea R., Herrera L.J., Angelov N., Paravina R.D. (2015). Color Range and Color Distribution of Healthy Human Gingiva: A Prospective Clinical Study. Sci. Rep..

[58] Caton J.G., Armitage G., Berglundh T., Chapple I.L.C., Jepsen S., Kornman K.S., Mealey B.L., Papapanou P.N., Sanz M., Tonetti M.S. (2018). A new classification scheme for periodontal and peri-implant diseases and conditions—Introduction and key changes from the 1999 classification. J. Clin. Periodontol..

[59] Kim D., Ahn S.-Y., Kim J., Park S.-H. (2017). Interrater and intrarater reliability of FDI criteria applied to photographs of posterior tooth-colored restorations. J. Prosthet. Dent..

[60] Tabatabaian F., Beyabanaki E., Alirezaei P., Epakchi S. (2021). Visual and digital tooth shade selection methods, related effective factors and conditions, and their accuracy and precision: A literature review. J. Esthet. Restor. Dent..

[61] Rosin M., Splieth C., Hessler M., Gärtner C., Kordaß B., Kocher T. (2002). Quantification of gingival edema using a new 3-D laser scanning method. J. Clin. Periodontol..

[62] Smith R., Lath D., Rawlinson A., Karmo M., Brook A. (2008). Gingival inflammation assessment by image analysis: Measurement and validation. Int. J. Dent. Hyg..

[63] Biesbrock A., Gibb R., Rubush M., Dunavent J., Gerlach R., Archila L. (2010). Concurrent clinical and image analysis assessment of gingivitis natural history. J Dent Res..

[64] Seshan H., Shwetha M. (2012). Gingival inflammation assessment: Image analysis. J. Indian Soc. Periodontol..

[65] Armitage G.C. (2004). The complete periodontal examination. Periodontology 2000.

[66] Campos L.A., Costa M.A., Bonafé F.S.S., Marôco J., Campos J.A.D.B. (2020). Psychosocial impact of dental aesthetics on dental patients. Int. Dent. J..

[67] Mancini L., Barootchi S., Thoma D.S., Jung R.E., Gallucci G.O., Wang H., Tavelli L. (2023). The peri-implant mucosa color: A systematic appraisal of methods for its assessment and clinical significance. Clin. Implant. Dent. Relat. Res..

[68] Carranza F., Newman M. (1998). Periodontología Clínica.

[69] Rauber G.B., Bernardon J.K., Vieira L.C.C., Baratieri L.N. (2017). Evaluation of a technique for color correction in restoring anterior teeth. J. Esthet. Restor. Dent..

[70] Lasserre J.-F., Pop-Ciutrila I.-S., Colosi H.-A. (2011). A comparison between a new visual method of colour matching by intraoral camera and conventional visual and spectrometric methods. J. Dent..

[71] He W., Park C.J., Byun S., Tan D., Lin C.Y., Chee W. (2020). Evaluating the relationship between tooth color and enamel thickness, using twin flash photography, cross-polarization photography, and spectrophotometer. J. Esthet. Restor. Dent..

[72] Armitage G.C. (1999). Development of a Classification System for Periodontal Diseases and Conditions. Ann. Periodontol..

[73] Katz M.S., Ooms M., Winnand P., Heitzer M., Bock A., Kniha K., Hölzle F., Modabber A. (2023). Evaluation of perfusion parameters of gingival inflammation using laser Doppler flowmetry and tissue spectrophotometry—a prospective comparative clinical study. BMC Oral Health.

[74] Hassan M.A., Amaral G.C.L.S.D., Saraiva L., Holzhausen M., Mendes F.M., Pannuti C.M., Stewart B., Malheiros Z.M., Benítez C., Nakao L.Y.S. (2025). Colorimetric analysis of intraoral scans: A novel approach for detecting gingival inflammation. J. Periodontol..

[75] Revilla-León M., Gómez-Polo M., Barmak A.B., Inam W., Kan J.Y., Kois J.C., Akal O. (2023). Artificial intelligence models for diagnosing gingivitis and periodontal disease: A systematic review. J. Prosthet. Dent..

[76] Shrivastava D., Srivastava K.C., Ganji K.K., Alam M.K., Al Zoubi I., Sghaireen M.G., Siciliano V.I. (2021). Quantitative Assessment of Gingival Inflammation in Patients Undergoing Nonsurgical Periodontal Therapy Using Photometric CIELab Analysis. BioMed Res. Int..

[77] Nagati R., Ragul M., Al-Qahtani N., Ravi K., Tikare S., Pasupuleti M. (2017). Clinical effec-tiveness of gingival depigmentation using conventional surgical scrapping and diode laser technique: A quasi experimental study. Glob. J. Health Sci..

[78] Abdel Moneim R., El Deeb M.A., Rabea A. (2020). Gingival pigmentation (cause, treatment and his-tological preview). Future Dent. J..

[79] Holmstrup P., Plemons J., Meyle J. (2018). Non–plaque-induced gingival diseases. J. Clin. Periodontol..

[80] Holmstrup P., Axéll T. (1990). Classification and clinical manifestations of oral yeast infections. Acta Odontol. Scand..

